# Predicting Variability and Reliability in Visual Field Testing: Short- and Long-Term Approaches

**DOI:** 10.1016/j.xops.2026.101065

**Published:** 2026-01-07

**Authors:** Jack Phu, Henrietta Wang, Jeremy C.K. Tan, Michael Kalloniatis

**Affiliations:** 1 School of Optometry and Vision Science, University of New South Wales, Kensington, New South Wales, Australia; 2 College of Optometry, University of Houston, Houston, Texas; 3 Department of Ophthalmology, Prince of Wales Hospital, Randwick, New South Wales, Australia; 4 Faculty of Medicine and Health, University of New South Wales, Kensington, New South Wales, Australia; 5 School of Medicine (Optometry), Deakin University, Waurn Ponds, Victoria, Australia

**Keywords:** Visual fields, Perimetry, Standard automated perimetry, 24-2, Frontloaded

## Abstract

**Purpose:**

To predict intrinsic measurement variability and reliability (any cause of data loss) in visual field (VF) results using a computer simulation model.

**Design:**

Computer simulation study.

**Subjects:**

One hundred thousand subjects simulated with empirical mean deviation, progression rate, variability, and reliability characteristics.

**Methods:**

One hundred thousand subjects were simulated to undergo 4 VF tests per visit, 3 monthly, over 20 years (long-term condition) and 4 VF tests per visit daily over 28 days (short-term condition). Permutations of 1-4 tests per visit over 3-, 6-, and 12-monthly (long-term) and 1-, 2-, and 4-daily (short-term) review intervals were used. Visual field variabilities were estimated sequentially until 3 consecutive visits returned variabilities within 5% of each other using a rolling window. The same was applied to reliability. The last visit of the window denoted the critical time to estimating variability using the consecutive clinical criterion (TcV) and critical time to estimating reliability using the consecutive clinical criterion (TcR) estimation. Additionally, we identified the critical time at which 3 consecutive visits were within 5% of the ground truth (critical time to estimating variability using the consecutive clinical criterion and comparison with the ground truth [TgV] and critical time to estimating reliability using the consecutive clinical criterion and comparison with the ground truth [TgR]).

**Main Measures:**

Critical time to estimating intrinsic variability and reliability.

**Results:**

The most intensive long-term approach (4 tests/visit, 3 monthly) required a median of 6 years to reach TcV. In the long-term, most subjects arrived at TcR within 2 years, but short-term testing (even with 1 test per visit) required only 5 days of daily testing. More tests per visit and more frequent reviews shortened the critical time. Average differences between the estimated variability and reliability at TcV and TcR and their ground truth results were clinically small (within 1 decibel and 10%, respectively). Mean deviation, progression rate, and variability were significant predictors of TcV and TgV for long-term follow-up, with no clinically significant predictors for short-term variability (R^2^ < 0.0001). Only reliability predicted TcR and TgR. Predictors had low coefficients of determination (<0.2).

**Conclusions:**

Longitudinal estimates of variability are not likely achievable in clinical practice, but short-term intensive VF testing unaffected by progression can return variability and reliability rates within reasonable timeframes. We provide a framework for the effect of variability for the likelihood of detecting differences in VF results over time, given reliability rates.

**Financial Disclosure(s):**

Proprietary or commercial disclosure may be found in the Footnotes and Disclosures at the end of this article.

Perimetry is a cornerstone in the assessment and monitoring of diseases of the visual pathway, especially glaucoma.^1^ Ideally, results of perimetric testing provide information on the functional status of the visual system. However, even with innovations in thresholding techniques, perimetric results suffer from imperfections that confound clinical interpretation. Broadly, these sources of imperfections in perimetry can be divided into variability and reliability. Variability refers to extrinsic or intrinsic factors that affect the returned threshold and its difference from the ground truth visual function.^2^ Variability represents expected fluctuations between measurements that do not represent true change. Sources include fatigue, learning, or distractions. Reliability refers to extrinsic or intrinsic factors that lead to data loss, that is, reasons for why the result needs to be discarded.^3^^,^^4^ Examples include errors in instrument setup, trigger-happy behavior, fatigue, or inattention.

Variability has been the topic of investigation since the inception of perimetry. In perimetry, psychophysical thresholding methods are limited by the practicalities of clinical testing and thus represent an approximation or estimation of the true underlying visual threshold. Obtaining the ground truth would require far more intensive testing and robust psychophysical approaches, such as method of constant stimuli, which are not practical for clinical use.^5^ Variability can be considered in various ways, such as: intravisit or intervisit; patient-related or test-/technique-/stimulus-related; and intrinsic or extrinsic.^1^ In clinical practice, it is essential to understand test variability to properly identify significant and true deviations in thresholds from normality, thus, identifying pathological change.^6^^,^^7^

Reliability has also been the subject of recent renewed research effort, in part, also stemming from more recent, faster test algorithms that trade speed and accuracy.^8^ In clinical practice, reliability refers to whether a result is accepted or discarded for clinical interpretation. Traditionally, reliability is frequently evaluated using catch trials, such as fixation losses, false-positives, and false-negatives, but the utility of these indices has been debated,^10^, ^11^, ^12^, ^9^ with other indicators of reliability having been suggested. Nonetheless, a concern is that data loss attributable to low test reliability reduces the ability of clinicians to detect disease progression.

Many studies suggest that an increased number of tests and test frequency can overcome issues pertaining to higher test variability and data loss from low reliability.^6^^,^^13^, ^14^, ^15^, ^16^, ^17^ However, a patient's variability and reliability may require many tests or visits to characterize, and therefore, whether they require more intensive follow-up would remain unknown until that point. This represents a challenge for resource allocation and personalization of care in clinical practice.

In the present study, we used a simulation model to generate a longitudinal series of visual field (VF) tests. Based on psychophysical theory that, despite their potential overlap, variability and reliability have a relatively consistent internal (or intrinsic) component (compared to variable extrinsic contributors) within individuals,^18^ we developed a model to identify the critical time point at which estimates of variability and reliability stabilize. As described earlier, intrinsic variability and reliability refer to the immutable component unique to the individual, and that which is difficult or impossible for the clinician to control (for example, fatigue and instructions represent extrinsic issues that could be affected by providing rest breaks and additional clarity, respectively). The first model was a long-term follow-up model, representing typical clinical practice of following patients over time. The second model was an intensive, short-term follow-up plan, representing a situation where potential disease- and age-related progression factors that may affect VF results are mitigated. We also performed regression analyses to identify baseline perimetric parameters that might affect the critical time point.

## Methods

### Design and Ethics Statement

This was a computer simulation study. Ethics approval was not required as patient data were not used for the simulation. Because the work did not use real human material or data, the Declaration of Helsinki was not applicable.

### Computer Simulation

We simulated a longitudinal VF series consisting of 100 000 patients. Each simulated patient was allocated a random baseline mean deviation value (sampled from a normal distribution with mean –4 decibls [dB] and standard deviation of 2 dB), progression rate (sampled from a skewed normal distribution with a starting location parameter of –0.05, scale of 0.8 and skew of –2), intrinsic intervisit variability (sampled from a log normal [to keep the results positive] distribution with mean 0.5 dB and sigma 0.4 dB), and intrinsic reliability (sampled from a beta distribution with low 0.5, high 0.99, alpha 9, and beta 1.5, which, in brief, represents a strong bias [alpha] toward more subjects with higher reliability [toward 0.99] and with a long left tail [beta] allowing a subset of subjects to fall toward the low end of reliability rate). In this context, “intrinsic” refers to a fundamental assumption that each patient uniquely and immutably has their own response criteria (as described earlier). Specifically, the progression rate and variability distributions were chosen to capture a diverse range of subjects, even though most patients in clinical practice are likely to be slow progressors in clinical practice (which would not provide a sufficiently wide dynamic range for the simulation).^19^, ^20^, ^21^ The reliability distribution was chosen to reflect that most patients are likely to return reliable results, with a smaller subset being less reliable.^15^

Each simulated patient had 4 VF tests simulated at each visit. For the long-term model, the follow-ups occurred at three-monthly intervals over 20 years. For the short-term model, follow-ups occurred daily over 28 days. Within each visit, 4 mean deviation results were generated, which were modulated by progression rate and variability. Then, for each result, reliability was assessed; if the intrinsic reliability threshold was not reached, the result was discarded, representing data loss. Though there are nuances in reliability and its interaction with test order,^15^ we aimed to characterize reliability in broad strokes, rather than under specific conditions. We also highlight the use of a unified reliability metric, rather than focusing on individual indices such as false-positives or false-negatives, serving to encapsulate different criteria that change across clinical or research settings and across time. For example, studies have used varying levels of false-positive rate as an indicator for reliability, and more recent studies have shown the imperfection with using this metric.^10^^,^^12^ Thus, the use of binarized reliability in the present study was a unified indicator to encompass a range of possible clinical circumstances.

The aforementioned represented the “core” data set. Using this complete series for each patient, we derived and assessed different combinations of conditions for comparison: number of tests per visit (1, 2, 3, or 4) over different follow-up intervals (3-monthly, 6-monthly, or yearly), that is, 12 permutations.

### Estimates of Intrinsic Variability

**Figure 1:**
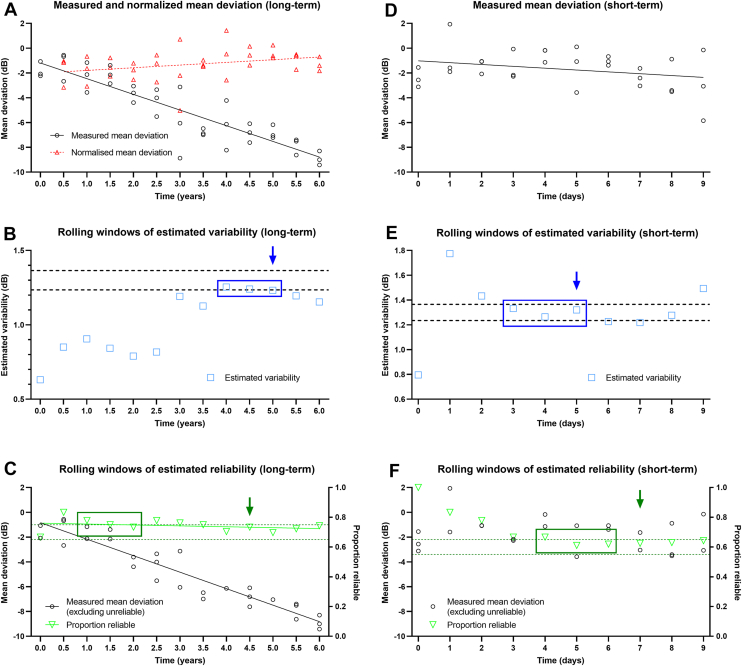
Approach to simulating longitudinal VF series to estimate variability and reliability demonstrated using a simulated subject. **A,** Mean deviation results (black circles) were generated at each visit and then normalized (red triangles) to account for progression rate (which was calculated at each visit) for the long-term follow-up plan. **B,** Using the data shown in (**A)**, variability (blue squares) is estimated at each visit. This occurs until 3 consecutive results are within 5% of each other (dark blue outline), with the last of the series taken as the critical time to consecutive variability estimation (TcV). Furthermore, this also occurs until 3 consecutive results are also within 5% of the ground truth (black dashed lines), with the last of the series taken as the critical time to the ground truth (TgV; dark blue arrow). **C,** The same data has its reliability estimated at each visit (green triangles). Using a rolling window, 3 consecutive reliability results that are within 5% is taken as the critical time to consecutive reliability estimation (TcR, dark green outline). The last of 3 consecutive results that are within 5% of the ground truth reliability (green dashed lines) is taken as the critical time to the ground truth (TgR; dark green arrow). **D,** The same mean deviation generation approach was generated using short-term follow-up, but with the assumption of no significant progression (and thus no normalization). **E and F,** demonstrate the rolling window approaches of variability and reliability estimation, respectively, for the short-term plan. dB = decibels; TcR = critical time to estimating reliability using the consecutive clinical criterion; TcV = critical time to estimating variability using the consecutive clinical criterion; TgR = critical time to estimating reliability using the consecutive clinical criterion and comparison with the ground truth; TgV = critical time to estimating variability using the consecutive clinical criterion and comparison with the ground truth; VF = visual field.

We calculated estimates of variability at each follow-up visit. For the long-term model, we first needed to account for any potential VF progression. Therefore, we first performed a linear regression analysis across all data points up until that follow-up visit to produce a slope representing the estimated progression rate. This was subtracted from the mean deviation values generated at that visit to obtain an estimate of “baseline” mean deviation unaffected by progression. This approach represented the clinical reality where ground truth progression rate is not knowable. Then, using all adjusted mean deviation values until that visit, we calculated variability which would then represent intrinsic variability. This represented the estimated variability at that visit (Fig 1A).

We applied a rolling window approach which required 3 consecutive visits to demonstrate variability estimates within 5% of each other (Fig 1B). In the rolling window, blocks of 3 consecutive visits were reviewed, until the consecutive criterion was met. This was to increase the robustness and ensure stability of the estimate. We regarded the last visit of the 3 consecutive estimates as the critical time to consecutive variability estimation (critical time to estimating variability using the consecutive clinical criterion [TcV]) (Fig 1B, dark blue outline). Note that if there were visits at which no valid result was obtained (due to low reliability), those visits were skipped for the purpose of counting consecutive results.

However, because there might be discordance between the magnitude of the estimated variability, even with the consecutiveness criterion, we also recorded the time point—critical time to estimating variability using the consecutive clinical criterion and comparison with the ground truth (TgV)—at which the estimated variability was within 5% of the ground truth variability (Fig 1B, dark blue arrow and dashed lines). This also had the same 3 consecutive visits requirement. Although ground truth variability is not conventionally clinically knowable, the purpose of TgV was to serve as a comparator for TcV. The difference between variability at TcV and the ground truth variability was also calculated to determine the degree of error when using estimate variability (which would be the case in most clinical situations). We emphasize that TgV (and critical time to estimating reliability using the consecutive clinical criterion and comparison with the ground truth [TgR] later) are not central metrics of interest in the study, but serve as a method to validate TcV and critical time to estimating reliability using the consecutive clinical criterion (TcR), which are estimated quantities.

### Estimates of Intrinsic Reliability

The approach to estimating intrinsic reliability was simpler. At each visit, we determined the totality of reliable VF test results until that point. This was the estimated reliability. Using a similar rolling window approach, we took the last of a consecutive series of 3 estimates of reliability that were within 5% of each other as the critical TcR (Fig 1C, dark green outline). Again, because of the potential discordance between estimated reliability and ground truth intrinsic reliability, we also recorded the time point—TgR—at which estimated reliability was within 5% of the ground truth reliability (Fig 1C, dark green arrow and dashed lines). Because intrinsic reliability is inherently difficult to measure clinically, we also calculated the difference between TcR and ground truth variability to determine the degree of error in the estimate.

Notably, one could argue that variable or noisy data at baseline affects subsequent estimations of critical time. This was the intention of the study. Because true variability and reliability are not known by the clinician, the need to obtain data from subsequent visits in a clinical scenario represents the methodologic approach of the study of estimating with the best available data.

### Short-Term Follow-Up Plans

For the short-term follow-up plan, because review intervals were short and occurred within 28 days (for example, a daily review would be 1/365th the progression rate), the normalization process through progression rate estimation was not performed. Instead, each data point obtained from each visit was used to obtain an estimate of variability at each visit (Fig 1D). The same rolling window approach was used to obtain TcV and TgV (Fig 1E). The same approach was applied for estimates of intrinsic reliability as was performed for the long-term model (Fig 1F).

### Baseline Variables Predicting Critical Time

Due to the nature of the simulation, we used progression rate, intrinsic variability and intrinsic reliability as potential predictors for critical time (TcV, TgV, TcR, and TgR). Because worse mean deviation results are associated with higher test–retest variability, they were not included as a potential independent variable for regression analysis. These relationships were first visualized using scatter plots and analyzed using linear regression analysis, before determining if higher order fittings were required.

### Statistical Analysis

We visualized critical time data using box-and-whisker distributions, which were analyzed using parametric or nonparametric descriptive statistics depending on whether they were normally distributed or not. Cumulative functions were generated with the proportion of subjects returning a valid critical time result as a function of time. We compared the critical time results across the various permutations of conditions. A *P* < 0.05 was considered statistically significant. We used GraphPad Prism version 9.4.0 (GraphPad Software) and custom written scripts on Sublime Text version 4 and enacted via Python 3.12 (Python Software Foundation) for data analysis and visualization.

## Results

The distributions (median and interquartile range) of baseline variables were as follows: mean deviation, –3.99 (–2.65, –5.35; encompassing mostly early stage glaucoma); slope –0.57 (–0.97 to –0.22); intrinsic variability 1.65 (1.26, 2.16); and intrinsic reliability 0.93 (0.89, 0.96) (Fig S1, available at www.ophthalmologyscience.org).

### Detection of Critical Time – Long-Term Model

The mean time and the distributions to detect TcV, TgV, TcR, and TgR are shown in Table 1 (and Fig S2, available at www.ophthalmologyscience.org). There was a wide distribution of possible detection times. Overall, increasing the number of tests per visit (*P* < 0.0001) and decreasing the review interval (*P* < 0.0001) resulted in shorter time to detection for TcV, TgV, and TgR. The critical times for detecting variability were higher than that of detection of reliability rates. Of concern, even the most intensive approach for testing (4 tests per year with 3 monthly visits) required a median time of 2.75 years to detect TcV, which was lengthened to 11 years for the least intensive approach (1 test per visit performed 12 monthly).

**Table 1:** Median and Interquartile Range of Critical Times (in Yrs) for Variability (TcV, TgV) and Reliability (TcR, TgR) for the Different Permutations of Review Period and Number of Tests per Visit for the Long-Term Follow-Up Plans

TcV	1 Test per Visit	2 Tests per Visit	3 Tests per Visit	4 Tests per Visit	*P* Value by Number of Tests
3 monthly	5.25 (2.75, 9)	3.75 (1.75, 7)	3 (1.5, 5.75)	2.75 (1.25, 5)	<0.0001
6 monthly	7.5 (4.5, 12)	6 (3.5, 10)	5 (3, 8.5)	5 (2.5, 8)	<0.0001
12 monthly	11 (7, 15)	10 (6, 14)	10 (6, 14)	10 (6, 13)	<0.0001∗
*P* value by review interval	<0.0001	<0.0001	<0.0001	<0.0001	

TgV	1 Test per Visit	2 Tests per Visit	3 Tests per Visit	4 Tests per Visit	*P* Value by Number of Tests
3 monthly	7 (3.75, 11)	5 (2.5, 8.5)	3.75 (1.75, 7.25)	3.25 (1.5, 6.25)	<0.0001
6 monthly	9 (5.5, 13)	7 (4, 11)	6 (3.5, 9.5)	5.5 (3, 8.5)	<0.0001
12 monthly	12 (8, 15)	11 (7, 14)	10 (7, 14)	10 (6, 14)	<0.0001
*P* value by review interval	<0.0001	<0.0001	<0.0001	<0.0001	

TcR	1 Test per Visit	2 Tests per Visit	3 Tests per Visit	4 Tests per Visit	*P* Value by Number of Tests
3 monthly	0.5 (0.5, 0.5)	0.5 (0.5, 1.25)	0.5 (0.5, 1.25)	0.75 (0.5, 1)	<0.0001
6 monthly	1 (1, 1)	1 (1, 2.5)	1 (1, 2.5)	1.5 (1, 2)	<0.0001
12 monthly	2 (2, 2)	2 (2, 5)	2 (2, 5)	3 (2, 4)	<0.0001
*P* value by review interval	<0.0001	<0.0001	<0.0001	<0.0001	

TgR	1 Test per Visit	2 Tests per Visit	3 Tests per Visit	4 Tests per Visit	*P* Value by Number of Tests
3 monthly	2.5 (0.5, 5)	1.75 (0.5, 3)	1.5 (1, 2.5)	1.25 (0.75, 2)	<0.0001
6 monthly	5 (1, 8.5)	3.5 (1, 6)	3 (2, 4.5)	2.5 (1.5, 4)	<0.0001
12 monthly	8 (2, 12)	6 (2, 10)	6 (2, 8)	4 (3, 7)	<0.0001
*P* value by review interval	<0.0001	<0.0001	<0.0001	<0.0001	

TcR = critical time to estimating reliability using the consecutive clinical criterion; TcV = critical time to estimating variability using the consecutive clinical criterion; TgR = critical time to estimating reliability using the consecutive clinical criterion and comparison with the ground truth; TgV = critical time to estimating variability using the consecutive clinical criterion and comparison with the ground truth.

∗ The result was statistically significant, despite each test condition have similar central tendency and distribution statistics.

In comparison, for TcR, most subjects had relatively short times to detection overall (median of 0.5-3 years across all conditions).

Given the prolonged duration to reaching TcV and TgV, we also repeated the simulation with a 10% (rather than 5%) tolerance for the rolling window analysis. As expected, the critical times were shorter (Table S1, available at www.ophthalmologyscience.org). However, even for the most intensive testing approach (4 tests per visit, 3 monthly), a median of 2.75 years were required for TcV, with a median of 8 years required if using a more commonly achieved approach of 1 test 12 monthly. There were minimal changes for TcR and TgR as the critical times were already low.

**Figure 2:**
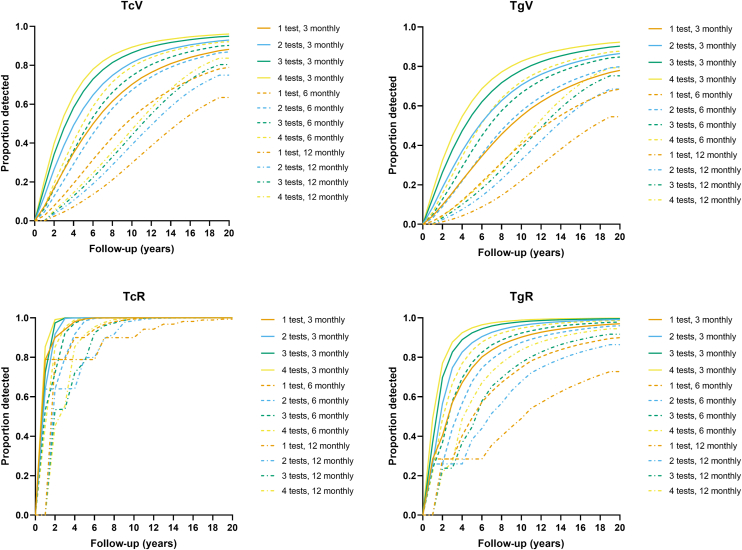
Cumulative distributions of proportion of subjects with TcV, TgV, TcR, or TgR detected as a function of follow-up time using the long-term follow-up plan. The different patterns of lines and colors indicate the permutations of number of tests and follow-up interval. TcR = critical time to estimating reliability using the consecutive clinical criterion; TcV = critical time to estimating variability using the consecutive clinical criterion; TgR = critical time to estimating reliability using the consecutive clinical criterion and comparison with the ground truth; TgV = critical time to estimating variability using the consecutive clinical criterion and comparison with the ground truth.

The proportions of cases with detected TcV, TgV, TcR, and TgR for each test and review interval permutation are shown in Figure 2. Again, more tests per session and shorter review intervals led to higher proportions of detected critical times. The difference between detection rates was marked when considering TcV and was less distinct for TcR. In other words, a lower number of tests that were spaced at 12 monthly intervals led to a marked decrease in detection rate for TcV.

In general, when using the long-term model, the critical time for estimations to be close to the ground truth (TgV and TgR) was less likely to be detected compared with estimations using the consecutive criterion (TcV and TcR).

### Detection of Critical Time – Short-Term Model

The short-term model results are shown in Table 2 and Figure S3 (available at www.ophthalmologyscience.org). Again, increasing number of tests per visit (*P* < 0.0001) and decreasing review interval (*P* < 0.0001) resulted in shorter time to detection for TcV, TgV, TcR, and TgR. Although this effect was statistically significant for TgV, TcR, and TgR (for example, median of 2 days was required for critical time estimation when performing daily tests across different numbers of tests), the differences were not likely clinically significant. Increasing the interval between visits increased the time required for detection, for example, increasing from a median of 2 days for daily testing to 4 days and 8 days for critical time estimation for every second day and every fourth day testing, respectively.

**Table 2:** Median and Interquartile Range of Critical Times (in Days) for Variability (TcV, TgV) and Reliability (TcR, TgR) for the Different Permutations of Review Period and Number of Tests per Visit for the Short-Term Follow-Up Plan

TcV	1 Test per Visit	2 Tests per Visit	3 Tests per Visit	4 Tests per Visit	*P* Value by Number of Tests
Daily	5 (3, 9)	4 (2, 6)	3 (2, 5)	3 (2, 5)	<0.0001
Every second day	10 (6, 16)	8 (4, 12)	6 (4, 10)	6 (4, 10)	<0.0001
Every fourth day	16 (8, 24)	12 (8, 20)	12 (8, 20)	12 (8, 16)	<0.0001
*P* value by review interval	<0.0001	<0.0001	<0.0001	<0.0001	

TgV	1 Test per Visit	2 Tests per Visit	3 Tests per Visit	4 Tests per Visit	*P* Value by Number of Tests
Daily	2 (2, 3)	2 (2, 2)	2 (2, 2)	2 (2, 2)	<0.0001
Every second day	4 (4, 6)	4 (4, 4)	4 (4, 4)	4 (4, 4)	<0.0001
Every fourth day	8 (8, 12)	8 (8, 8)	8 (8, 8)	8 (8, 8)	<0.0001
*P* value by review interval	<0.0001	<0.0001	<0.0001	<0.0001	

TcR	1 Test per Visit	2 Tests per Visit	3 Tests per Visit	4 Tests per Visit	*P* Value by Number of Tests
Daily	2 (2, 2)	2 (2, 4)	2 (2, 3)	2 (2, 3)	<0.0001
Every second day	4 (4, 4)	4 (4, 8)	4 (4, 6)	4 (4, 6)	<0.0001
Every fourth day	8 (8, 8)	8 (8, 16)	8 (8, 12)	8 (8, 12)	<0.0001
*P* value by review interval	<0.0001	<0.0001	<0.0001	<0.0001	

TgR	1 Test per Visit	2 Tests per Visit	3 Tests per Visit	4 Tests per Visit	*P* Value by Number of Tests
Daily	2 (2, 7)	2 (2, 5)	3 (2, 4)	2 (2, 4)	<0.0001
Every second day	4 (4, 12)	4 (4, 8)	4 (4, 8)	4 (4, 8)	<0.0001
Every fourth day	8 (8, 8)	8 (8, 16)	8 (8, 16)	8 (8, 16)	<0.0001
*P* value by review interval	<0.0001	<0.0001	<0.0001	<0.0001	

TcR = critical time to estimating reliability using the consecutive clinical criterion; TcV = critical time to estimating variability using the consecutive clinical criterion; TgR = critical time to estimating reliability using the consecutive clinical criterion and comparison with the ground truth; TgV = critical time to estimating variability using the consecutive clinical criterion and comparison with the ground truth.

**Figure 3:**
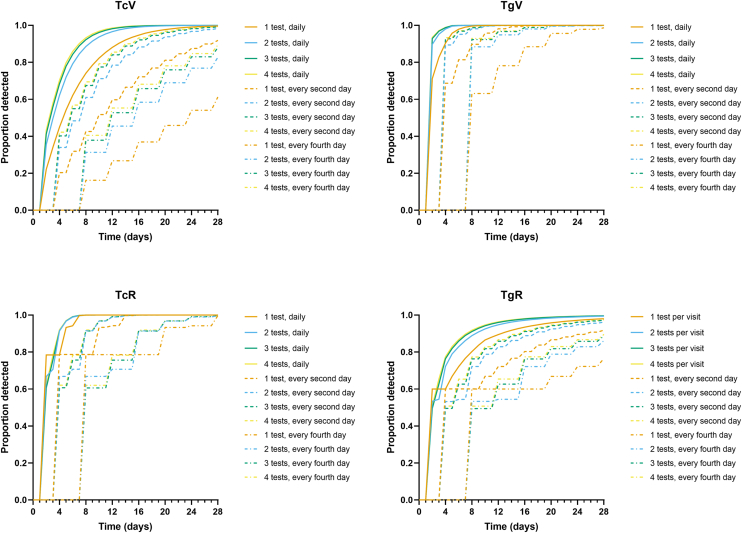
Cumulative distributions of proportion of subjects with TcV, TgV, TcR, or TgR detected as a function of follow-up time using the short-term follow-up plan. The different patterns of lines and colors indicate the permutations of number of tests and follow-up interval. TcR = critical time to estimating reliability using the consecutive clinical criterion; TcV = critical time to estimating variability using the consecutive clinical criterion; TgR = critical time to estimating reliability using the consecutive clinical criterion and comparison with the ground truth; TgV = critical time to estimating variability using the consecutive clinical criterion and comparison with the ground truth

The cumulative proportion of cases with detected TcV, TgV, TcR, and TgR for each test and review interval permutation are shown in Figure 3. As alluded to in Table 2, performing >3 or 4 tests per visit offered limited benefit compared with 2 tests per visit for most conditions. Note that despite the “shorter” critical time for TgV compared with TcV, TgV is not clinically measurable as the ground truth is not likely knowable given the nature of imperfect clinical data.

### Difference in Critical Detection Time: Consecutive Criterion and Ground Truth

**Figure 4:**
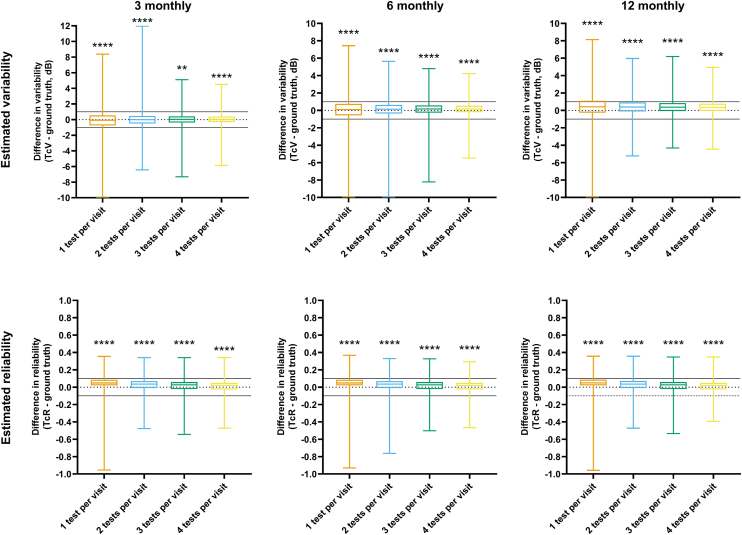
Difference in estimates of variability (TcV) and reliability (TcR) from the respective ground truth values for each review interval and number of tests per visit using the long-term follow-up plan. The box-and-whiskers represent the median, interquartile range, and full range. The black dotted line indicates no difference (y = 0), and the solid lines indicate a difference of 1 dB and 0.1 for variability and reliability, respectively. The asterisks indicate level of statistical significance for a 1-sample t-test (∗∗, *P* < 0.01; ∗∗∗∗, *P* < 0.0001). dB = decibels; TcR = critical time to estimating reliability using the consecutive clinical criterion; TcV = critical time to estimating variability using the consecutive clinical criterion.

**Figure 5:**
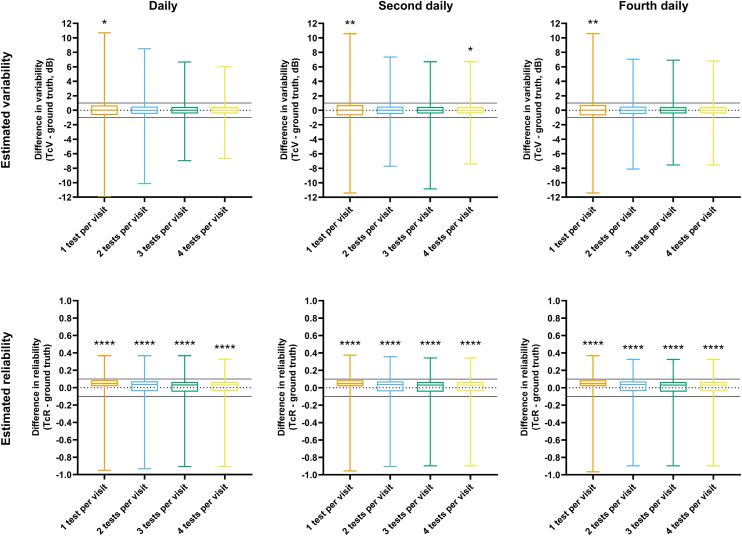
Difference in estimates of variability (TcV) and reliability (TcR) from the respective ground truth values for each review interval and number of tests per visit using the short-term follow-up plan. The box-and-whiskers represent the median, interquartile range, and full range. The black dotted line indicates no difference (y = 0), and the solid lines indicate a difference of 1 dB and 0.1 for variability and reliability, respectively. The asterisks indicate level of statistical significance for a 1-sample t-test (∗, *P* < 0.05; ∗∗, *P* < 0.01; ∗∗∗∗, *P* < 0.0001). dB = decibels; TcR = critical time to estimating reliability using the consecutive clinical criterion; TcV = critical time to estimating variability using the consecutive clinical criterion

Where a subject had both TcV and TgV pairs or TcR and TgR pairs, the difference between variability or reliability at the critical time with the consecutive criterion (which could be clinically measured) and the ground truth (which is generally not clinically observable) were plotted in Figures 4 and 5 for long-term and short-term conditions, respectively. The difference plot revealed a bias toward critical time with the consecutive criterion (TcV and TcR) being higher than the ground truth (TgV and TgR, respectively) for most test and review interval permutation. In other words, in most instances, TcV provided a slight overestimate of variability and TcR returned a slightly overestimate of reliability. Over half of the differences of TcV were within 1 dB of the ground truth for 3 monthly and 6 monthly review intervals. The 12 monthly review interval led to a systematically overestimated variability, but the median values were still <0.5 dB. Similarly, over half of the differences of TcR were within 0.1 of the ground truth. An increasing number of tests performed per visit resulted in a smaller mean difference and narrower distribution of values. Increasing the follow-up interval marginally increased the difference in variability but had a minimal effect on the difference in reliability.

For the short-term schedule, there was a slight bias toward the consecutive criterion measurement to be slightly higher than the ground truth in variability (TcV), but this was not likely to be clinically significant (differences of 0.01 dB with 1 test per visit performed daily and 4 tests per visit performed daily, with 1 test per visit performed every 4 days). The differences in reliability were also statistically significant (all conditions *P* < 0.0001), but the magnitude of differences were also small and not likely clinically significant (range of differences of 0.01 to 0.05).

Overall, these results suggested that whilst statistically significant differences existed, TcV and TcR—essentially clinically observable estimates of critical time—could serve as reasonable estimates of the ground truth within <0.5 dB and within 10%, respectively.

### Regression Analysis for Predictors of Critical Time

The effects of baseline parameters (mean deviation, slope, intrinsic variability, intrinsic reliability), the number of tests per visit, and follow-up interval as independent variables for regression analysis for critical time are shown in Table 3 and Figures S4 to S7 (available at www.ophthalmologyscience.org). For TcV and TgV, all variables were significant predictors (*P* < 0.0001) except for intrinsic reliability (*P* = 0.2513 for TcV and *P* = 0.4757 for TgV). A worse (more negative) baseline mean deviation, faster (more negative) progression rate, lower intrinsic variability, more tests per visit, and shorter follow-up intervals were associated with a shorter critical time to estimated variability. However, the sum of the coefficients of determination of the predictors for TcV and TgV were 0.2768 and 0.3053, respectively, indicating that only approximately 28% to 30% of the variance was accounted for by these predictors.

**Table 3:** Regression Analysis Results of Baseline Variables Contributing to Critical Time Outcomes for the Long-Term Follow-Up Schedule

	Coefficient	Standard Error	*P* Value	R^2^
TcV				
Baseline mean deviation (dB)	0.8219	0.0072	<0.0001	0.1159
Progression rate (dB/yr)	1.861	0.0266	<0.0001	0.0469
Intrinsic variability (dB)	2.200	0.0195	<0.0001	0.1140
Intrinsic reliability	0.2758	0.3011	0.2513	<0.0001
TgV				
Baseline mean deviation (dB)	0.9154	0.0079	<0.0001	0.1227
Progression rate (dB/yr)	1.865	0.0296	<0.0001	0.0397
Intrinsic variability (dB)	2.699	0.0213	<0.0001	0.1429
Intrinsic reliability	0.2344	0.3286	0.4757	<0.0001
TcR				
Baseline mean deviation (dB)	–0.0019	0.0015	0.2118	<0.0001
Progression rate (dB/yr)	–0.0039	0.0053	0.4625	<0.0001
Intrinsic variability (dB)	–0.0085	0.0040	0.0343	<0.0001
Intrinsic reliability	–7.005	0.0545	<0.0001	0.1416
TgR				
Baseline mean deviation (dB)	0.0036	0.0059	0.5353	<0.0001
Progression rate (dB/yr)	–0.0194	0.0209	0.3542	<0.0001
Intrinsic variability (dB)	0.0124	0.0158	0.4314	<0.0001
Intrinsic reliability	–24.84	0.2176	<0.0001	0.1153

dB = decibels; TcR = critical time to estimating reliability using the consecutive clinical criterion; TcV = critical time to estimating variability using the consecutive clinical criterion; TgR = critical time to estimating reliability using the consecutive clinical criterion and comparison with the ground truth; TgV = critical time to estimating variability using the consecutive clinical criterion and comparison with the ground truth.

For TcR and TgR, intrinsic reliability was a significant predictor (*P* < 0.0001). Progression rate was a statistically significant predictor of TcR (*P* = 0.0343), but the coefficient of determination was negligible (<0.0001). A higher intrinsic reliability was associated with a shorter critical time to estimated reliability. However, the coefficients of determination of reliability remained low at 0.1416 for TcR and 0.1153 for TgR.

The regression results for the short-term schedule are shown in Table 4 and Figures S8 to S11 (available at www.ophthalmologyscience.org). None of the factors predicted TcV or TgV. For TcR, only intrinsic reliability was a significant predictor (*P* < 0.0001), with a low coefficient of determination of 0.147. Notably, baseline progression rate had no effect as it was not incorporated into the short-term model. Intrinsic reliability was also a significant predictor of TgR, but again accounted for <20% of variance (*P* < 0.0001, R^2^ = 0.188). Although baseline mean deviation and progressors were significant (*P* = 0.0065 and *P* = 0.0032, respectively), both had a negligible coefficient of determination of <0.0001.

**Table 4:** Regression Analysis Results of Baseline Variables Contributing to Critical Time Outcomes Using Short-Term Follow-Up Data

	Coefficient	Standard Error	*P* Value	R^2^
TcV				
Baseline mean deviation (dB)	–0.0001	0.0029	0.818	<0.0001
Progression rate (dB/yr)	–0.0009	0.0115	0.9346	<0.0001
Intrinsic variability (dB)	–0.0099	0.0077	0.2006	<0.0001
Intrinsic reliability	0.1094	0.1125	0.3312	<0.0001
TgV				
Baseline mean deviation (dB)	0.0004	0.0006	0.5502	<0.0001
Progression rate (dB/yr)	–0.0065	0.0026	0.011	<0.0001
Intrinsic variability (dB)	0.0013	0.0018	0.4369	<0.0001
Intrinsic reliability	0.016	0.025	0.5223	<0.0001
TcR				
Baseline mean deviation (dB)	–0.0011	0.001	0.2732	<0.0001
Progression rate (dB/yr)	–0.0026	0.004	0.5178	<0.0001
Intrinsic variability (dB)	–0.0020	0.0027	0.4572	<0.0001
Intrinsic reliability	–8.4444	0.0367	<0.0001	0.117
TgR				
Baseline mean deviation (dB)	0.0016	0.0031	0.6109	<0.0001
Progression rate (dB/yr)	–0.0077	0.0126	0.5415	<0.0001
Intrinsic variability (dB)	–0.0063	0.0085	0.4578	<0.0001
Intrinsic reliability	–35.45	0.1112	<0.0001	0.204

dB = decibels; TcR = critical time to estimating reliability using the consecutive clinical criterion; TcV = critical time to estimating variability using the consecutive clinical criterion; TgR = critical time to estimating reliability using the consecutive clinical criterion and comparison with the ground truth; TgV = critical time to estimating variability using the consecutive clinical criterion and comparison with the ground truth.

## Discussion

Our results showed that it would take an impractical number of years of follow-up to obtain estimates of intrinsic perimetric variability using observed VF data obtained at regular review intervals via a long-term follow-up plan, beyond that which is likely to be clinically useful. Instead, short-term intensive VF testing can return estimates of intrinsic perimetric variability, within 5 days if performing daily testing, or by 4 days if performing 2 tests per visit daily. The critical time can be tailored by modulating either the interval of testing or the number of tests per visit. Our results show that 5 test results—as the median time using 1 test per visit—would result in 50% of cases of variability being estimated. Intrinsic reliability is estimable using either long-term or short-term approaches, generally within a short VF series, occurring in 50% of cases by the third visit.

### Intrinsic Variability

Based on signal detection theory, intrinsic variability represents the internal noise component that varies across individuals.^18^ This is notably different to extrinsic factors such as stimulus parameters and the testing environment.^22^ Signal-to-noise ratio has been used for examining stimulus characteristics mediating defect detection.^23^^,^^24^ A patient with high variability returns low fidelity, noisy data, potentially leading to false-positive or false-negative interpretation.

Clinically observable variability is challenging to appreciate in long-term VF series, as there are further confounders of perimetric learning and potential functional progression, in addition to the effects of age.^25^^,^^26^ Even using clinically observable TcV as an approximation of TgV, a common clinical paradigm of performing 1 VF test every 12 months^27^^,^^28^ led to a median critical time of 14 to 15 years to estimate reliability using this criterion. Frontloading VF tests (2 tests per visit 6 monthly)^13^^,^^29^ shortened the critical time to 9.5 to 11 years,^15^ but the benefits were modest for improving estimates of variability.

This result suggests that estimation of variability over conventional long-term clinical review periods requires an impractically long follow-up period, due to the requirement of “normalization” of the mean deviation at each follow-up visit to account for potential progression. Instead, intensive short-term review may facilitate variability estimation. There are some benefits of multiple tests per visit, with frontloading with 2 tests per eye per visit as an appropriate strategy for this purpose. Previous work has not suggested a significant effect of fatigue on perimetry results; in fact, the converse of the second result tending to be more reliable is often found.^13^^,^^29^ Our present work suggests that there is a plateau in the benefit at 3 tests per visit, which has not been widely evaluated in clinical practice.

The concept of performing multiple trials within a clinical visit or session for estimating variability is not new. Older perimeters and thresholding algorithms, such as the Humphrey Field Analyzer's full threshold program, had the option of obtaining a metric quantifying short-term fluctuations.^30^^,^^31^ Using the Humphrey Field Analyzer full threshold program as an example, this option assesses several test locations twice during the same test, returning 2 threshold values with which to provide an estimate of a patient's intratest variability. However, the full threshold program has a prohibitively long test duration that is impractical for routine clinical testing and has been replaced in clinical settings by Bayesian approaches such as the Swedish Interactive Thresholding Algorithm (SITA) family.^8^

From a clinical perspective, it is therefore necessary to balance the number of tests per visit with test interval; more tests per visit mean fewer visits required to return an estimate of variability. Either way, substantial clinic time in the short-term is required. Portable technologies that facilitate out-of-office testing, including home monitoring, may be leveraged for this purpose.^32^, ^33^, ^34^, ^35^, ^36^ Platforms such as tablet perimetry or virtual reality headsets have been shown to be correlated with standard automated perimetry, acceptable to patients, with modest success in adherence outside of clinical settings.^37^^,^^38^ However, due to differences in dynamic range and technical specifications, variability characteristics generated by each device may not be interchangeable.^39^ Furthermore, many previous studies mainly report on adherence for once-weekly or once-monthly testing,^32^^,^^33^^,^^35^ with some emerging evidence for the feasibility of more intensive testing, as per our simulation model.^34^^,^^36^

### Intrinsic Reliability

Reliability (i.e., the rate of data loss) is important for the accumulation of sufficient data for clinical interpretation and progression analysis.^14^^,^^40^^,^^41^ Estimations of intrinsic reliability were more readily achievable using either long- or short-term approaches. Most cases had reliability estimates returned by the third visit (e.g., by 2 years with 12-monthly follow-up). Interestingly, there was little benefit of performing more tests per visit for estimating TcR (i.e., clinically observable reliability data); the benefits of more tests per visit was only seen for TgR, due to the need for greater precision.

This result suggests that clinicians would be able to understand a patient's intrinsic reliability within 3 visits in most cases using clinically observable data alone. A clinical question of a significant “threshold” for defining good or poor levels of reliability remains (and hence whether to aim for TcR or TgR for reliability estimation). The value judgment and practical implications of a threshold for poor intrinsic reliability require further investigation to define acceptable levels of reliability. The question of reliability and usable data is nuanced, as there may be a spectrum of potentially useful clinical data hidden within test results not meeting traditional reliability criteria.^9^^,^^10^

### Factors Affecting the Critical Time

Factors affecting critical time were more significant for the long-term model, but not for the short-term model. Notably, this included progression rate and relevant surrogates of measurement dynamic range (mean deviation). Again, this highlights the ability of short-term intensive review to mitigate their effects to facilitate estimation of variability.

Both the number of tests and follow-up intervals are surrogates for data volume. However, the present study focused on visits, rather than per-test instances. In other words, we did not “count” the absolute number of tests in terms of the consecutive criterion. Thus, the incremental benefit shortening critical time when performing greater numbers of tests per visit was small. However, the overall coefficients of determination were low, showing that predicting critical time using baseline variables is not practical or useful, with high interindividual variation and interactive effects.

### Clinical Implications

Clinical guidelines often recommend the establishment of a “baseline” for patients for future progression analysis, typically by having a larger number of tests early in the follow-up period, followed by a tailored approach later.^15^ Although this baseline set of results serves as a comparator for future follow-ups, it also has the potential to characterize variability for a more tailored approach to testing. Supposing that extrinsic factors, such as pupil size/light adaptation, stimulus parameters, background illumination, and test algorithm,^22^ are adequately controlled, we can examine the “minimal” effect of intrinsic variability on the likelihood of detection of a signal using signal-to-noise ratio and binomial probability equations. Given an example goal of detecting a difference of 0.8 dB (such as progression; “signal”), we can map out the probability of detection on ≥1 visit given 1, 2, 3, or 4 tests per visit across various levels of variability (“noise”) (Table 5). With incrementally increasing levels of variability, the probability of detection decreases, thus requiring a greater number of tests per visit to achieve similar probabilities.

**Table 5:** Probability of Detecting a Difference of 0.8 dB in ≥1 Visit, Given Different Levels of Variability and Given 1–4 Visual Field Tests Performed per Visit

Variability (dB)	Signal-To-Noise Ratio (1/Variability)	1 Test per Visit	2 Tests per Visit	3 Tests per Visit	4 Tests per Visit
0.5	1.6	0.9452	0.9974	0.9998	0.99996
1	0.8	0.7881	0.9468	0.9864	0.9955
1.5	0.533	0.7031	0.9086	0.9691	0.9867
2	0.4	0.6554	0.874	0.9628	0.9855
2.5	0.32	0.6255	0.844	0.9371	0.9685

dB = decibels.

Therefore, given the ability to estimate variability, we could potentially tailor a testing approach to optimize resource allocation. A patient with very low variability (e.g., 0.5 dB) may not require or benefit from high volume testing. On the other hand, greater variability (e.g., >1.5 dB) would likely benefit from a frontloaded or clustered approach. There may also be circumstances where variability is so high that perimetric testing is unfeasible for meaningful detection. Furthermore, integrating data loss due to low test variability provides additional insight regarding time required to detect a signal of interest, that is, sensitivity change given a specific level of noise (variability). Using this model, we have created a reference application to derive these probabilities (Fig S12, available at www.ophthalmologyscience.org; weblink to.exe Supplementary File and github link to calculator: https://github.com/jackphu/visual_fields_public.git).

Currently, estimations of observable clinical variability require calculation by a technician or clinician, which is labor-intensive. We propose that future methods to calculate patient variability using sequential data could be integrated into existing perimetric data management software to reduce the human burden.

### Limitations

As a modeling and simulation study, the present work relied on several assumptions and used fixed distributions of key perimetric parameters. The purpose of this was to elicit the potential effects of each baseline parameter and to illustrate the concept of critical time. We also used a simple linear regression model, as per routine clinical methods. Future models could incorporate more complex interactions between parameters and other regression approaches in an exploratory manner.

We purposefully decoupled variability and reliability. In practice, they may be related to each other; a patient who has a variable result may also be likely to return low reliability results. However, the simulation considered reliability in general terms of data loss, rather than representing results that have been excluded due to high variability. The concept of data loss has been a topic of recent interest, with studies highlighting nuance in traditionally “unreliable” data points that may still have clinical utility for progression analysis.^10^^,^^42^^,^^43^ A notable advantage of a simulation study (rather than real data) that is agnostic to a specific reliability metric is that the model (and its results) are adaptable to paradigm shifts in reliability indicators. For example, current manufacturer specifications of the Humphrey field analyzer flag false-positive rates >15% as unreliable, despite evidence to the contrary. In future, this cutoff could be re-evaluated and a model that uses a unified or agonist reliability metric would be able to be adapted to this change.

Our model used a simulated distribution consisting mostly of early glaucoma. This is likely most patients seen in many clinical practices, but more importantly, this group represents a clinical conundrum of appropriate testing intensity. In cases of more advanced glaucoma, it is commonplace to closely follow and reassess patients, but with limited resources, the question is how frequently earlier or milder cases of glaucoma should be monitored. In future, a study on more advanced cases of glaucoma, perhaps also including information from the 10-2 test grid or equivalent, would provide further insights.

Finally, as a computer simulation model, these results require empirical testing with data obtained from human participants in clinical settings. To fully test this model would require a large, comprehensive longitudinal study with intensive perimetric testing. Such an experiment would require significant resources and likely cooperation amongst several groups to achieve. Though challenging, technologies such as portable perimetry offer methods that may facilitate more intensive testing, as demonstrated by recent studies.^32^^,^^35^^,^^36^

### Conclusions

In conclusion, short-term intensive testing, such as a clustered or frontloaded approach, that is not affected by potential disease- or age-related progression can return estimates of intrinsic perimetric variability, which is an important factor for determining the likelihood of detecting changes in the VF. Intensive testing may be achievable with current home monitoring technologies. Estimation of intrinsic reliability is likely achievable by the third visit for many patients. This work further emphasizes the need for a tailored approach to perimetric testing, and we provide a simple framework and application that could be used in clinical practice.
