# Supplementary material for: Predicting Variability and Reliability in Visual Field Testing: Short- and Long-Term Approaches
Source: Ophthalmol Sci. 2026 Jan 7;6(3):101065. doi: 10.1016/j.xops.2026.101065 (PMC12907646; doi:10.1016/j.xops.2026.101065)
Supplement: Figures S1–S12 and Table S1 [file mmc1.pdf]

Supplementary: Predicting variability and reliability in visual field testing: short- and long-term approaches

Jack Phu[1,2,3,4], Henrietta Wang[1,2], Jeremy C. K. Tan[5,6], Michael Kalloniatis[1,7,8]

[1] School of Optometry and Vision Science, University of New South Wales, Kensington, New South Wales, Australia

[2] Centre for Eye Health, University of New South Wales, Kensington, New South Wales, Australia

[3] Faculty of Medicine and Health, University of Sydney, Sydney, NSW

[4] Concord Clinical School, Concord Repatriation General Hospital, Concord, NSW

[5] Department of Ophthalmology, Prince of Wales Hospital, Randwick, New South Wales, Australia

[6] Faculty of Medicine and Health, University of New South Wales, Kensington, NSW, Australia

[7] University of Houston College of Optometry, Houston, TX

[8] School of Medicine (Optometry), Deakin University, Waurn Ponds, Victoria, Australia

Number of Figures: 12 Number of Tables: 1

Corresponding Author: Jack Phu

Address for reprints: School of Optometry and Vision Science, Gate 14 Barker Street Rupert Myers Building South Wing, University of New South Wales Sydney 2052, New South Wales, Australia

Email: jack.phu@unsw.edu.au

Keywords: visual fields; perimetry; standard automated perimetry; 24-2; frontloaded

Financial support: The work was supported, in part, by an NHMRC Ideas Grant to MK and JP (1186915). The funding organisation had no role in the design or conduct of this research.

Conflict of interest: No conflicting relationship exists for any author.

Running head: Variability and reliability in perimetry

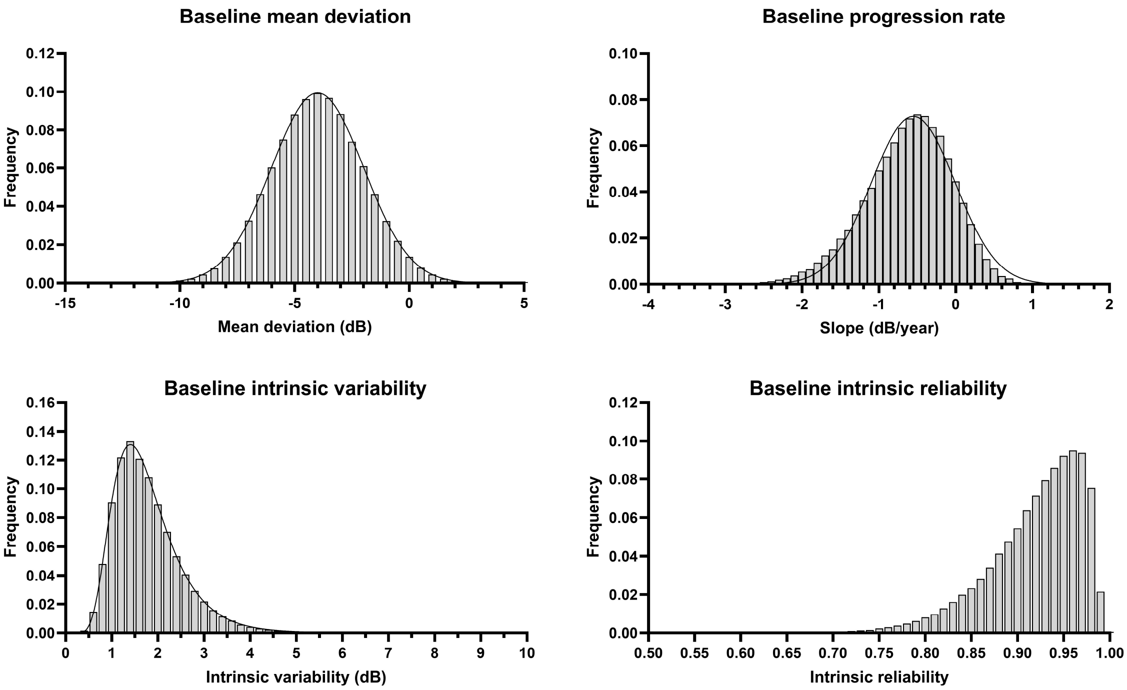

Supplementary Figure 1: Frequency distributions of baseline characteristics of the simulated cohort.

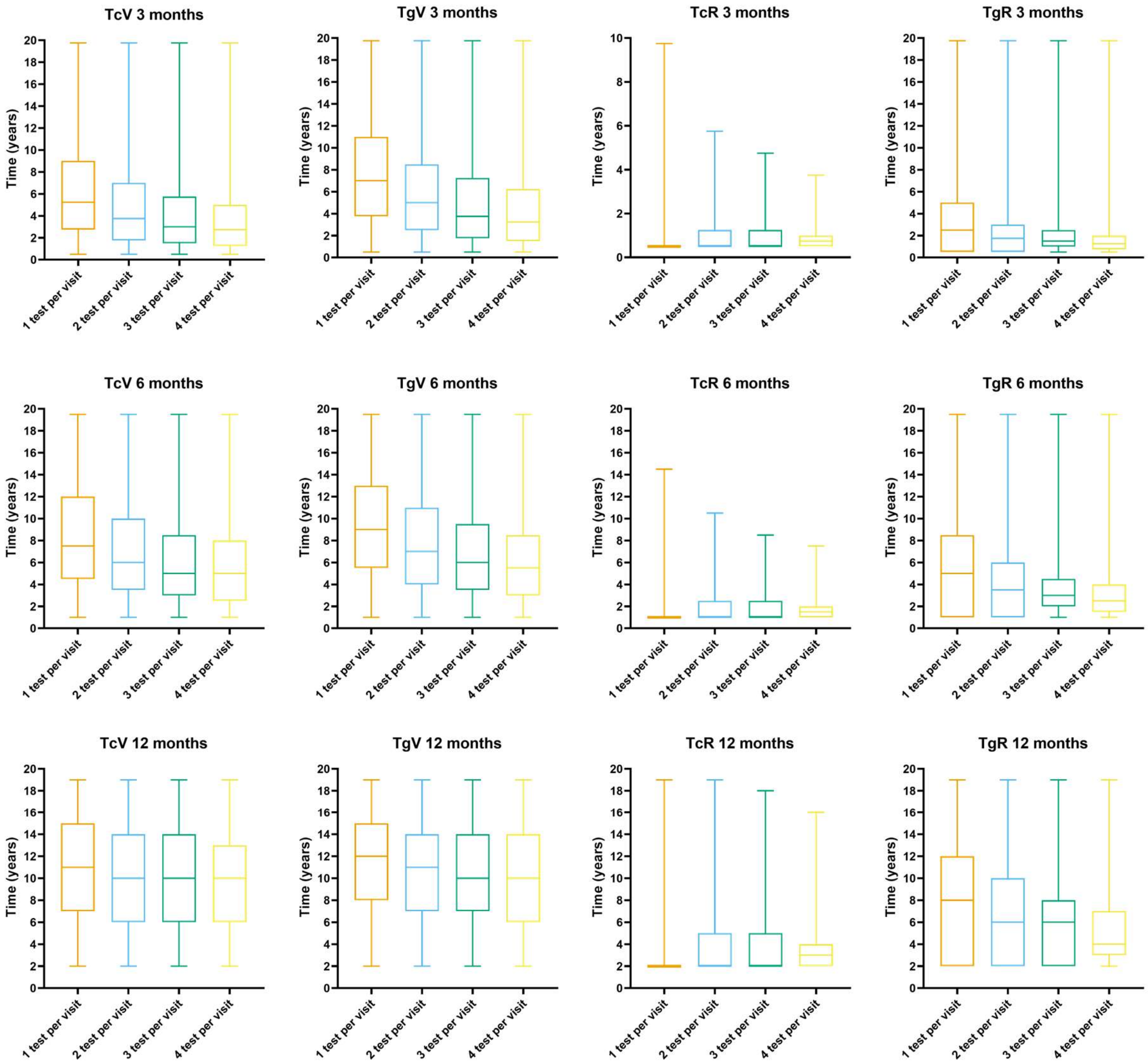

Supplementary Figure 2: Distribution of critical times (TcV, TgV, TcR and TgR) in years for each follow up interval (3 months, 6 months or 12 months) and by number of tests per visit. The box-and-whiskers indicate the median, interquartile range and full range.

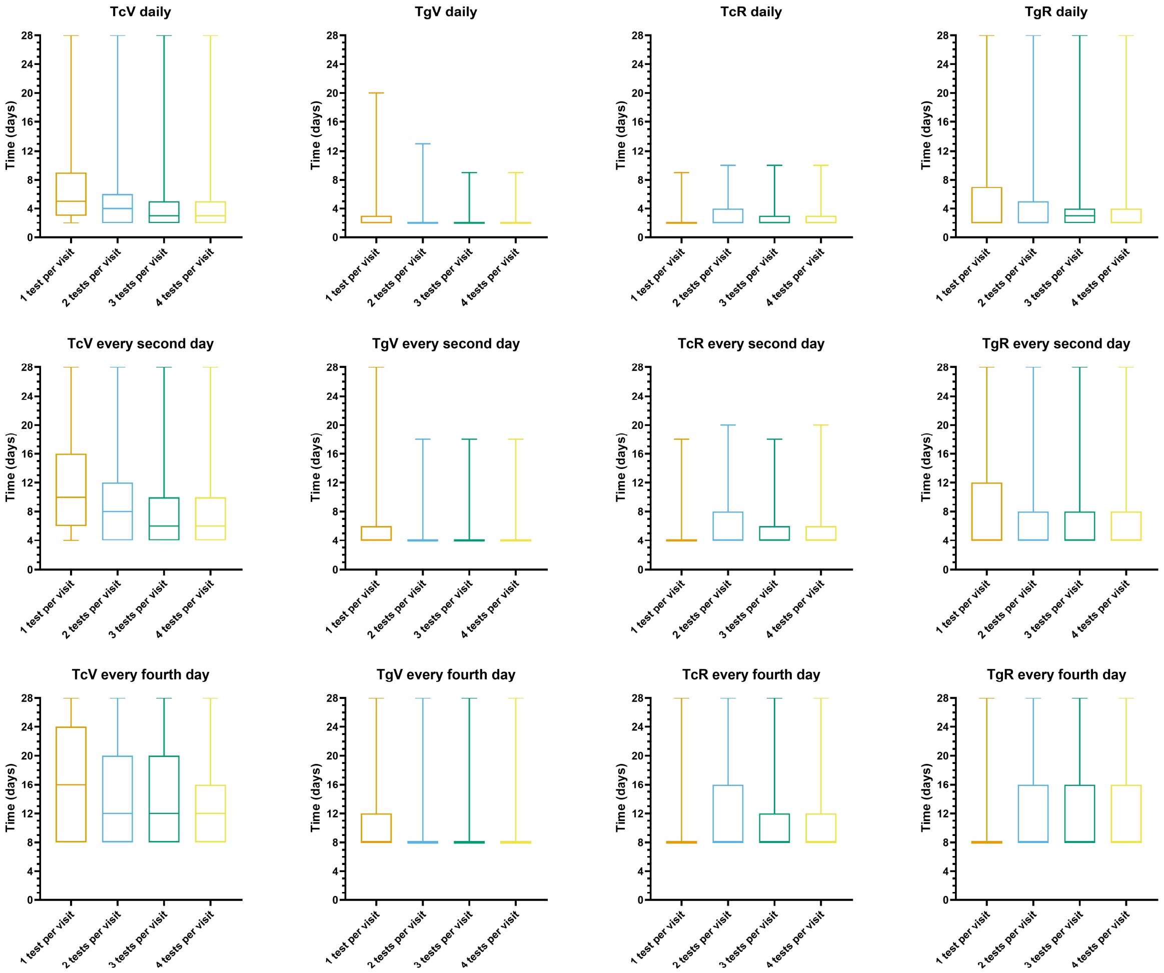

Supplementary Figure 3: Distribution of critical times (TcV, TgV, TcR and TgR) in days for each follow up interval (daily, every second day and every fourth day) and by number of tests per visit. The box-and-whiskers indicate the median, interquartile range and full range.

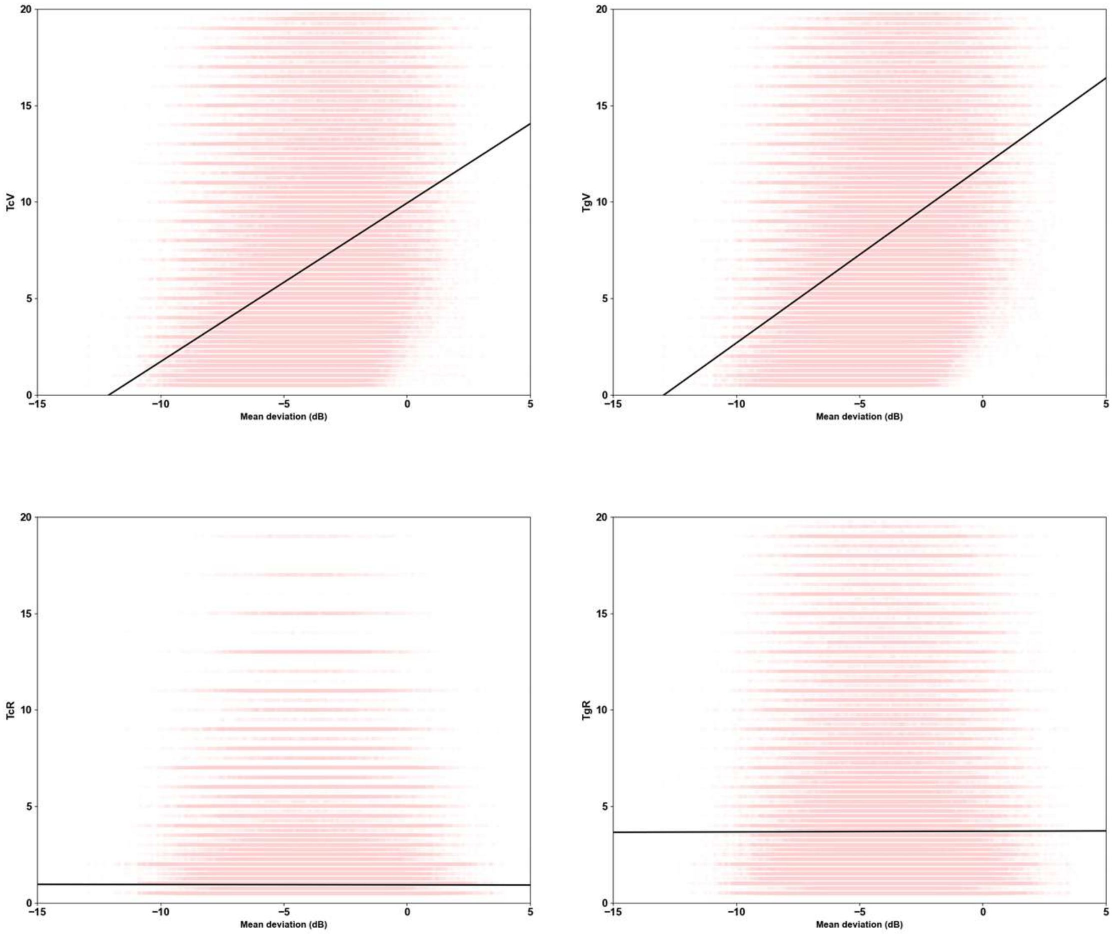

Supplementary Figure 4: Critical time (TcV, TgV, TcR and TgR) as a function of baseline mean deviation (dB) using long-term follow-up data. Each red dot represents a simulated patient’s result, and the black solid line indicates the regression analysis (see Table 2 for regression results).

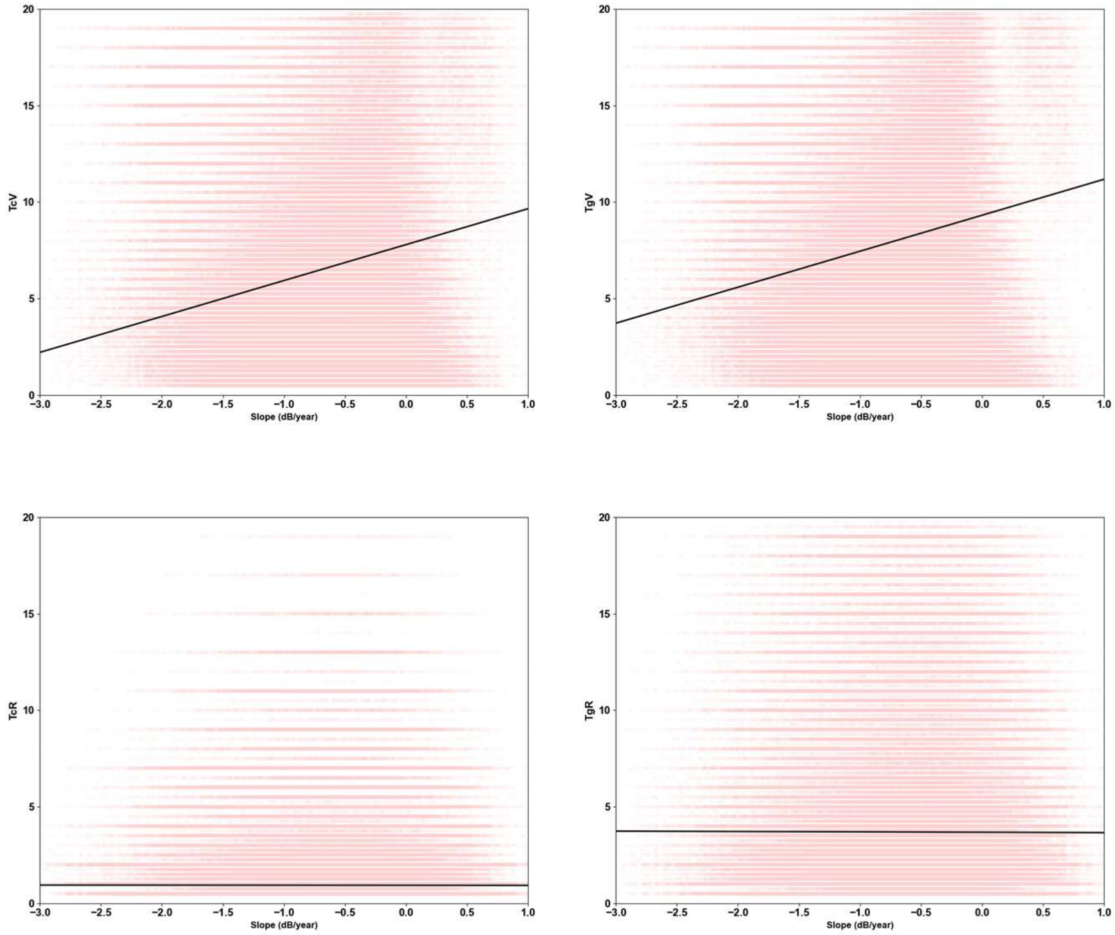

Supplementary Figure 5: Critical time (TcV, TgV, TcR and TgR) as a function of baseline progression rate (dB/year) using long-term follow-up data. Each red dot represents a simulated patient's result, and the black solid line indicates the regression analysis (see Table 2 for regression results).

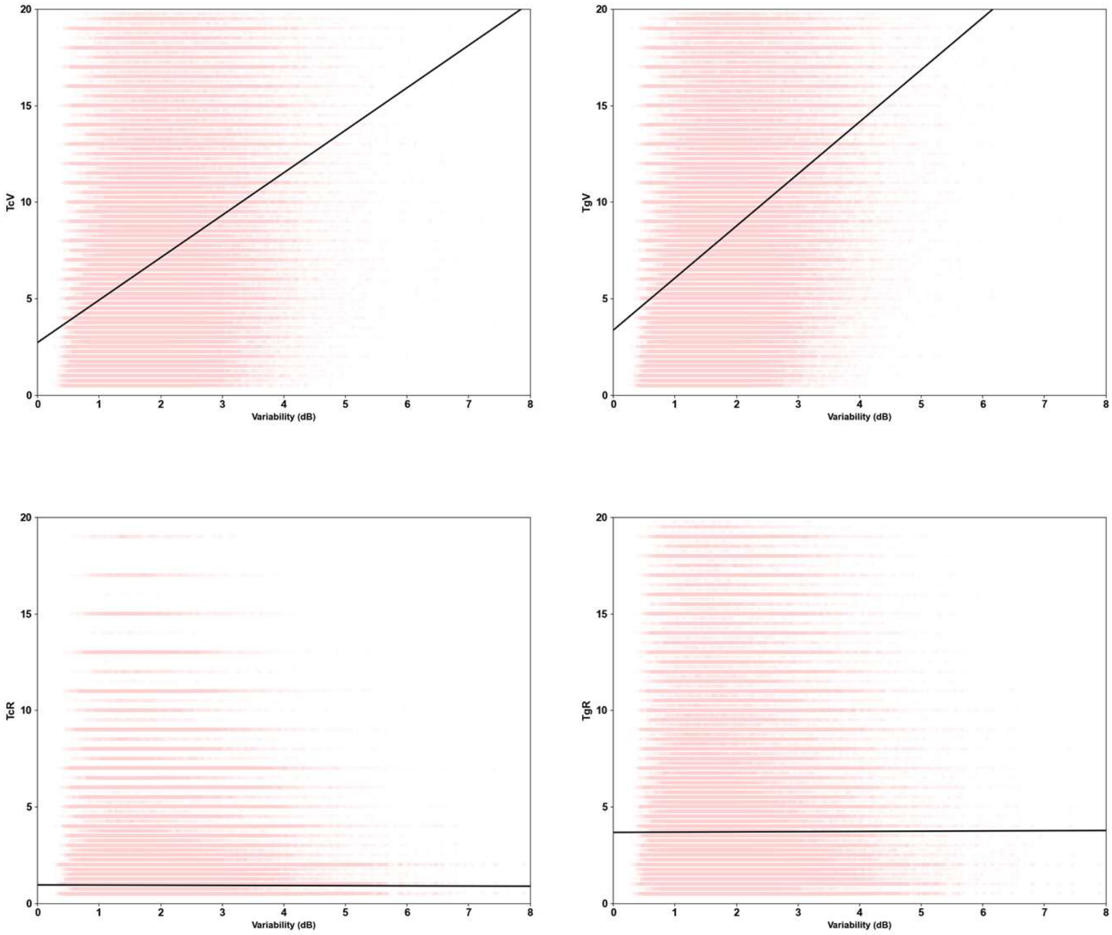

Supplementary Figure 6: Critical time (TcV, TgV, TcR and TgR) as a function of intrinsic variability (dB) using long-term follow-up data. Each red dot represents a simulated patient's result, and the black solid line indicates the regression analysis (see Table 2 for regression results).

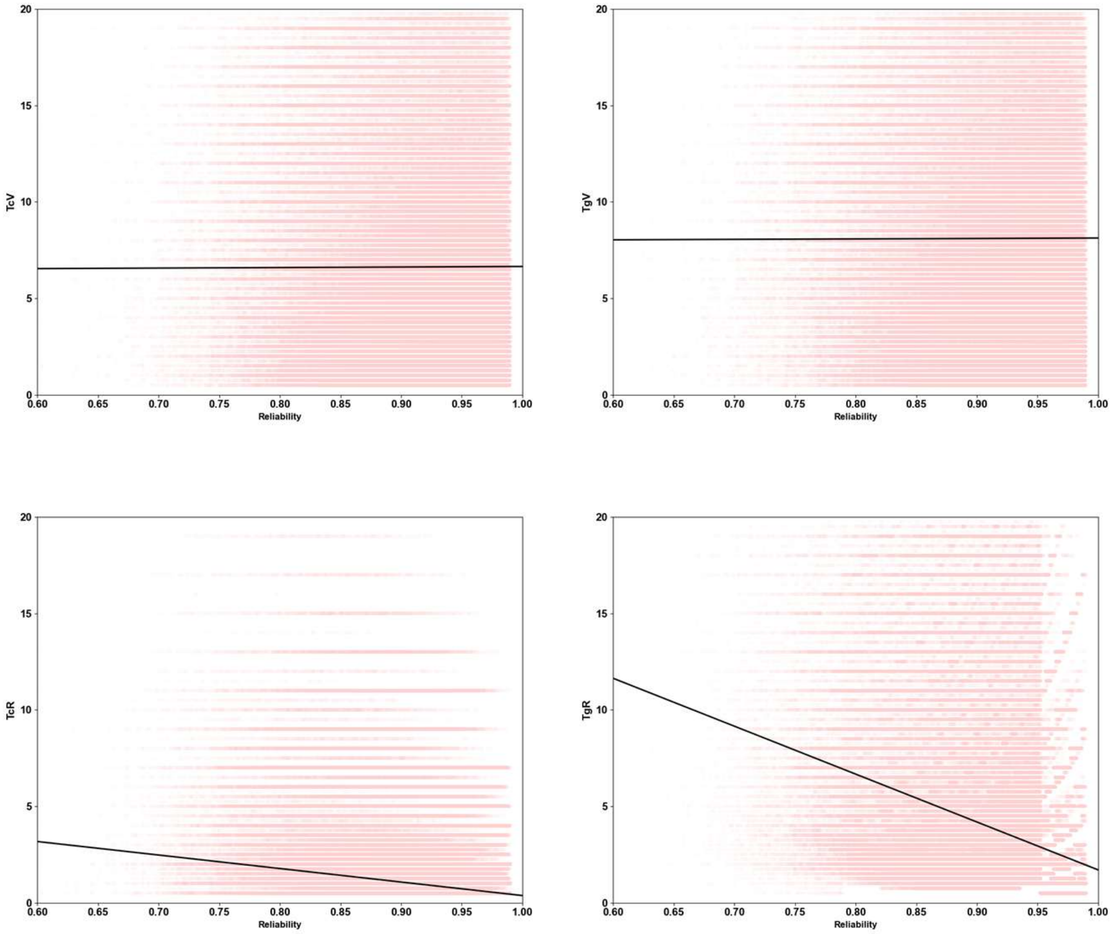

Supplementary Figure 7: Critical time (TcV, TgV, TcR and TgR) as a function of intrinsic reliability using long-term follow-up data. Each red dot represents a simulated patient's result, and the black solid line indicates the regression analysis (see Table 2 for regression results).

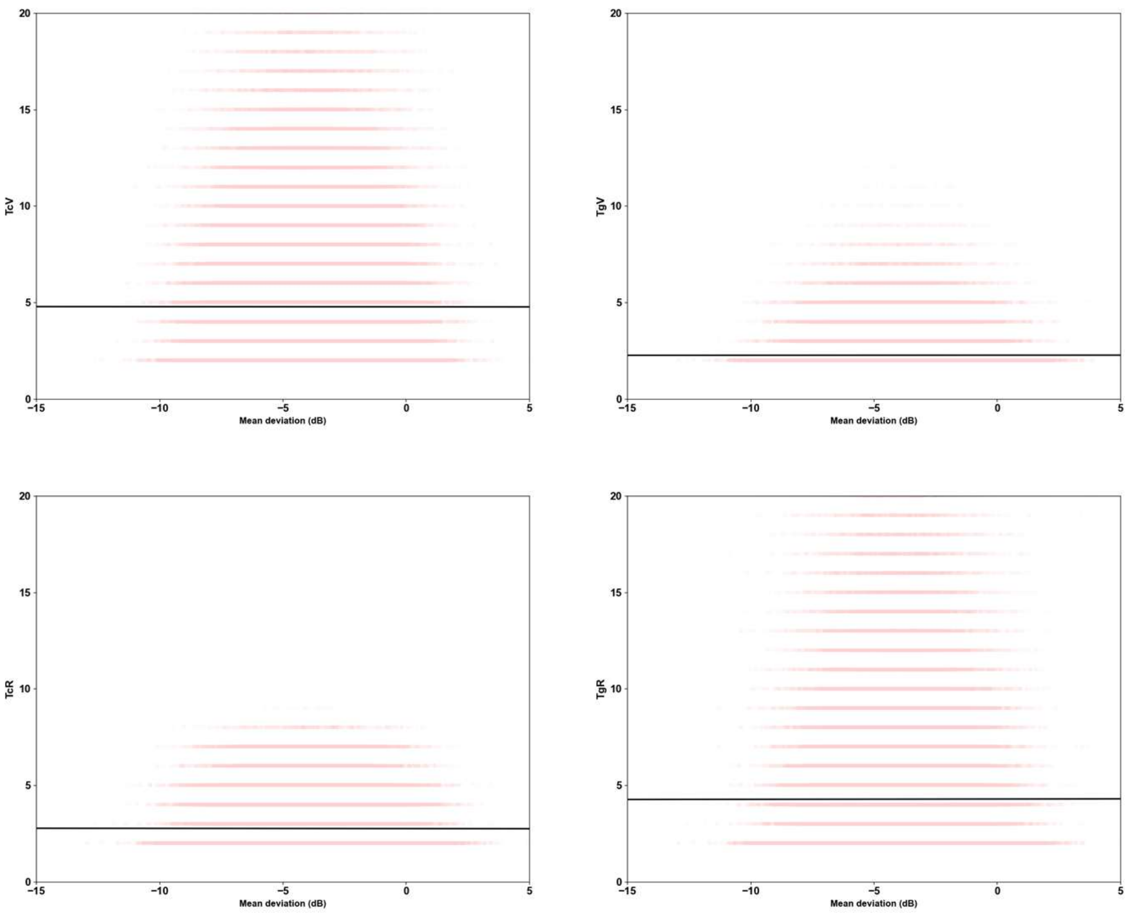

Supplementary Figure 8: Critical time (TcV, TgV, TcR and TgR) as a function of baseline mean deviation (dB) using short-term follow-up data. Each red dot represents a simulated patient's result, and the black solid line indicates the regression analysis (see Table 3 for regression results).

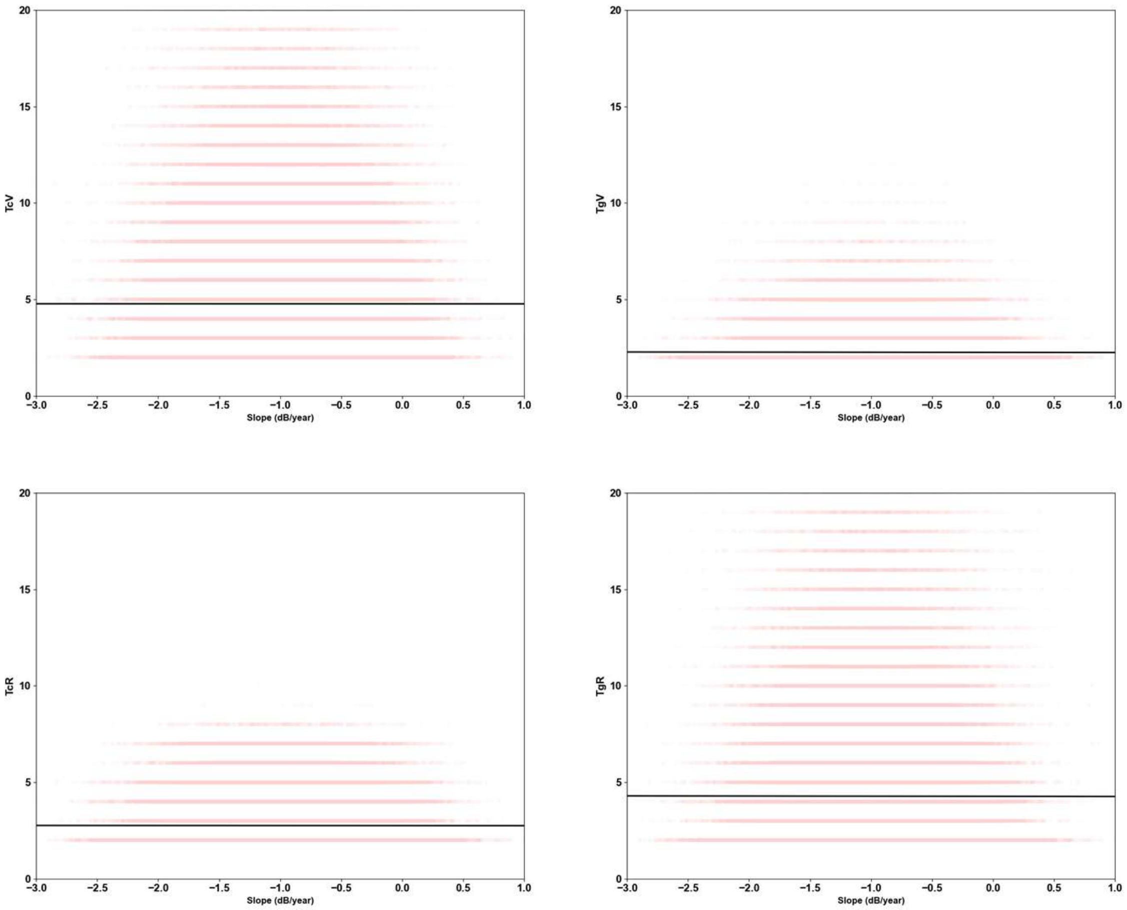

Supplementary Figure 9: Critical time (TcV, TgV, TcR and TgR) as a function of baseline progression rate (dB/year) using short-term follow-up data. Each red dot represents a simulated patient's result, and the black solid line indicates the regression analysis (see Table 3 for regression results).

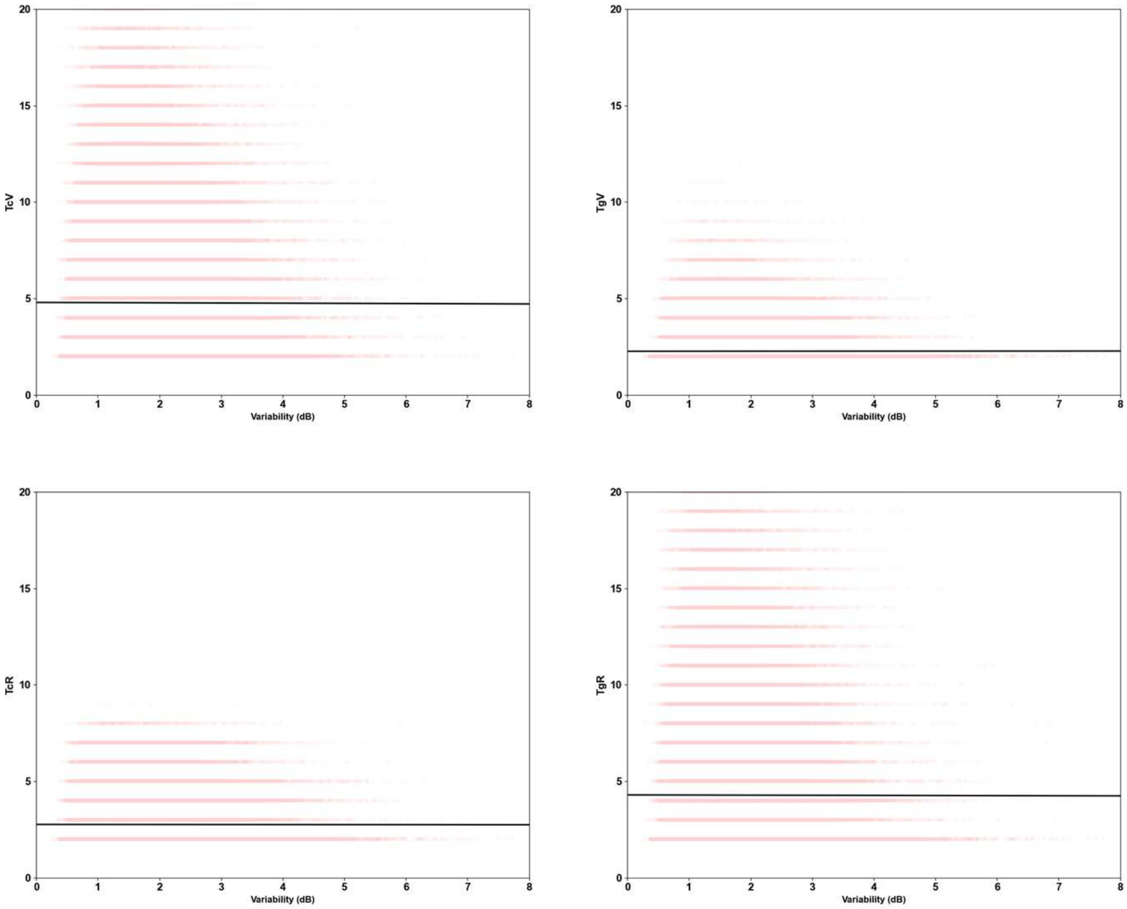

Supplementary Figure 10: Critical time (TcV, TgV, TcR and TgR) as a function of intrinsic variability (dB) using short-term follow-up data. Each red dot represents a simulated patient's result, and the black solid line indicates the regression analysis (see Table 3 for regression results).

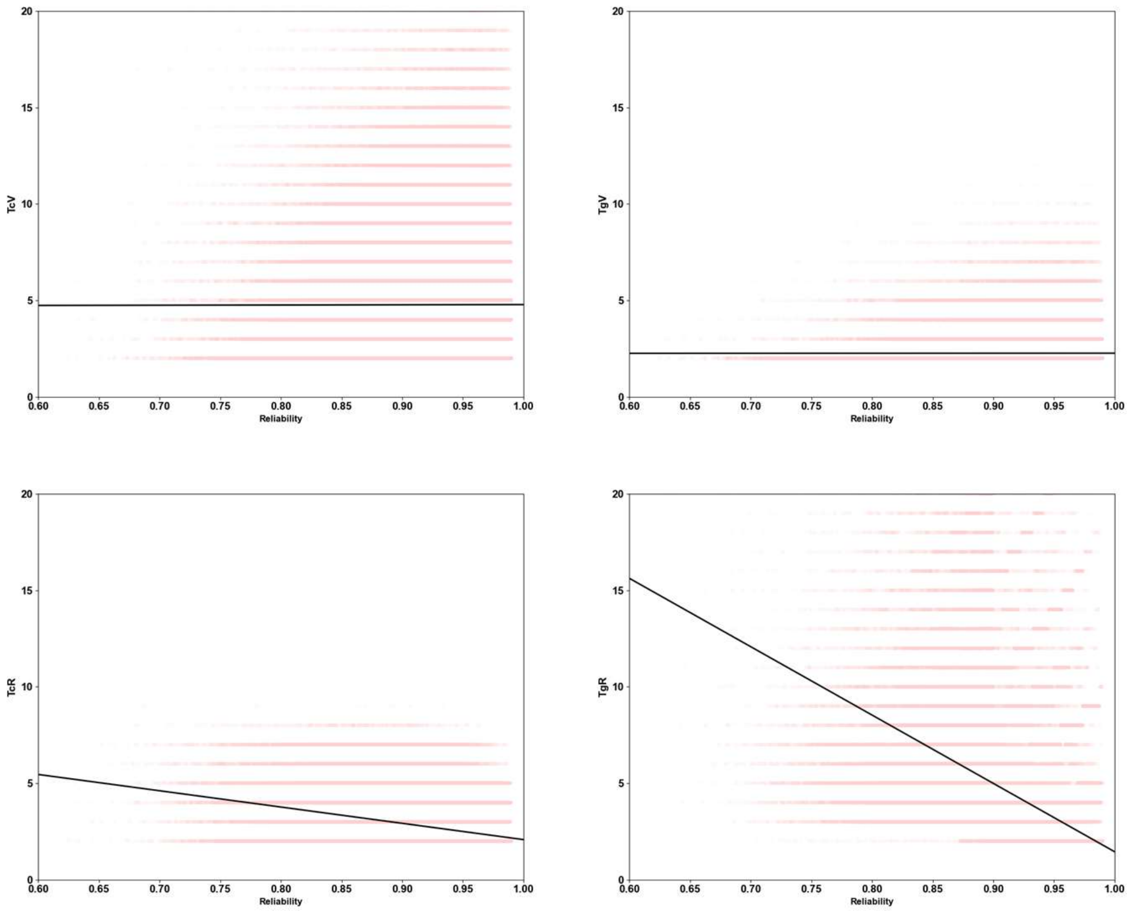

Supplementary Figure 11: Critical time (TcV, TgV, TcR and TgR) as a function of intrinsic reliability using short-term follow-up data. Each red dot represents a simulated patient's result, and the black solid line indicates the regression analysis (see Table 3 for regression results).

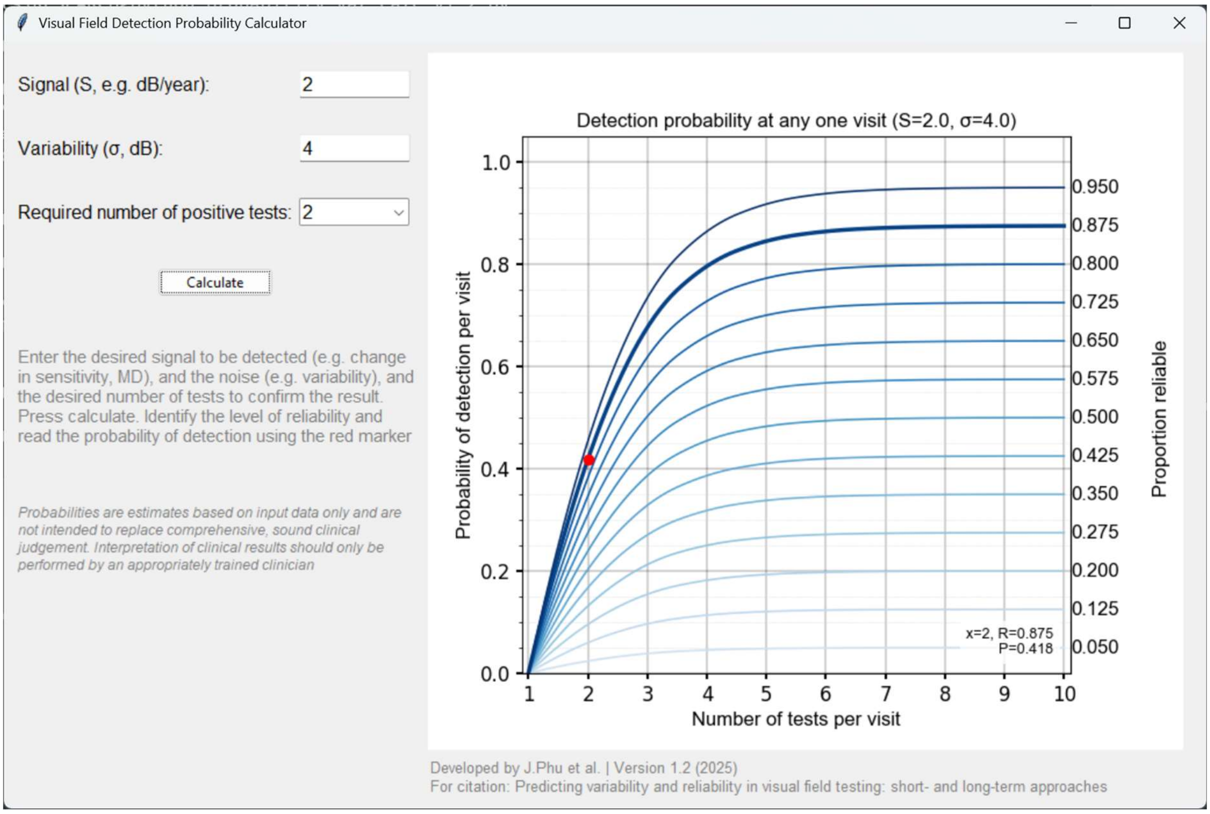

Supplementary Figure 12: A screenshot from an application that may be used for estimating detection probability at any one visit given a desired signal (e.g. progression rate, change in sensitivity), noise (variability), and required number of positive tests. Each different shade of blue line indicates levels of reliability, with darker blues indicating higher reliability. Probability of detection is indicated by the left y-axis and the red marker.

Supplementary Table 1: Median and interquartile range of critical times (in years) for variability (TcV, TgV) and reliability (TcR, TgR) for the different permutations of review period and number of tests per visit when using a modified 10% tolerance for the rolling window for the long-term model.

TcV	1 test per visit	2 tests per visit	3 tests per visit	4 tests per visit	P-value by number of tests
3 monthly	5.25 (2.75, 9)	3.75, (1.75, 7)	3 (1.5, 5.75)	2.75 (1.25. 5)	<0.0001
6 monthly	7.5 (4.5, 112)	6 (3.5 (10)	5 (3, 8.5)	5 (2.5, 8)	<0.0001
12 monthly	11 (7, 15)	10 (6, 14)	10 (6, 14)	10 (6, 13)	<0.0001
P-value by review interval	<0.0001	<0.0001	<0.0001	<0.0001	
TgV	1 test per visit	2 tests per visit	3 tests per visit	4 tests per visit	P-value by number of tests
3 monthly	7 (3.75, 11)	5 (2.5, 8.5)	3.75 (1.75, 7.25)	3.25 (1.5, 6.25)	<0.0001
6 monthly	9 (5.5, 13)	7 (4, 11)	6 (3.5, 9.5)	5.5 (3, 8.5)	<0.0001
12 monthly	12 (8, 15)	11 (7, 14)	10 (7, 14)	10 (6, 14)	<0.0001
P-value by review interval	<0.0001	<0.0001	<0.0001	<0.0001	
TcR	1 test per visit	2 tests per visit	3 tests per visit	4 tests per visit	P-value by number of tests
3 monthly	0.5 (0.5, 0.5)	0.5 (0.5, 1.25)	0.5 (0.5, 1.25)	0.75 (0.5, 1)	<0.0001
6 monthly	1 (1, 1)	1 (1, 2.5)	1 (1, 2.5)	1.5 (1, 2)	<0.0001
12 monthly	2 (2, 2)	2 (2, 5)	2 (2, 5)	3 (2, 4)	<0.0001
P-value by review interval	<0.0001	<0.0001	<0.0001	<0.0001	
TgR	1 test per visit	2 tests per visit	3 tests per visit	4 tests per visit	P-value by number of tests
3 monthly	2.5 (0.5, 5)	1.75 (0.5, 3)	1.5 (1, 2.5)	1.25 (0.75, 2)	<0.0001
6 monthly	5 (1, 8.5)	3.5 (1, 6)	3 (2, 4.5)	2.5 (1.5, 4)	<0.0001
12 monthly	8 (2, 12)	6 (2, 10)	6 (2, 8)	4 (3, 7)	<0.0001
P-value by review interval	<0.0001	<0.0001	<0.0001	<0.0001	
